# Monitoring gait in multiple sclerosis with novel wearable motion sensors

**DOI:** 10.1371/journal.pone.0171346

**Published:** 2017-02-08

**Authors:** Yaejin Moon, Ryan S. McGinnis, Kirsten Seagers, Robert W. Motl, Nirav Sheth, John A. Wright, Roozbeh Ghaffari, Jacob J. Sosnoff

**Affiliations:** 1 Department of Kinesiology and Community Health, University of Illinois at Urbana-Champaign, Urbana, Illinois, United States of America; 2 Department of Electrical and Biomedical Engineering, University of Vermont, Burlington, Vermont, United States of America; 3 MC10 Inc., Lexington, Massachusetts, United States of America; 4 Department of Physical Therapy, University of Alabama at Birmingham, Birmingham, Alabama, United States of America; Saint Louis University, UNITED STATES

## Abstract

**Background:**

Mobility impairment is common in people with multiple sclerosis (PwMS) and there is a need to assess mobility in remote settings. Here, we apply a novel wireless, skin-mounted, and conformal inertial sensor (BioStampRC, MC10 Inc.) to examine gait characteristics of PwMS under controlled conditions. We determine the accuracy and precision of BioStampRC in measuring gait kinematics by comparing to contemporary research-grade measurement devices.

**Methods:**

A total of 45 PwMS, who presented with diverse walking impairment (Mild MS = 15, Moderate MS = 15, Severe MS = 15), and 15 healthy control subjects participated in the study. Participants completed a series of clinical walking tests. During the tests participants were instrumented with BioStampRC and MTx (Xsens, Inc.) sensors on their shanks, as well as an activity monitor GT3X (Actigraph, Inc.) on their non-dominant hip. Shank angular velocity was simultaneously measured with the inertial sensors. Step number and temporal gait parameters were calculated from the data recorded by each sensor. Visual inspection and the MTx served as the reference standards for computing the step number and temporal parameters, respectively. Accuracy (error) and precision (variance of error) was assessed based on absolute and relative metrics. Temporal parameters were compared across groups using ANOVA.

**Results:**

Mean accuracy±precision for the BioStampRC was 2±2 steps error for step number, 6±9ms error for stride time and 6±7ms error for step time (0.6–2.6% relative error). Swing time had the least accuracy±precision (25±19ms error, 5±4% relative error) among the parameters. GT3X had the least accuracy±precision (8±14% relative error) in step number estimate among the devices. Both MTx and BioStampRC detected significantly distinct gait characteristics between PwMS with different disability levels (p<0.01).

**Conclusion:**

BioStampRC sensors accurately and precisely measure gait parameters in PwMS across diverse walking impairment levels and detected differences in gait characteristics by disability level in PwMS. This technology has the potential to provide granular monitoring of gait both inside and outside the clinic.

## 1. Introduction

Multiple sclerosis (MS) is an immune-mediated disease that affects an estimated 400,000 people in the USA, and has a worldwide prevalence of 2.5M [1]. MS is characterized by inflammatory demyelination and axonal damage in the central nervous system, which result in conduction delays and blockage of electrical potentials along neuronal pathways [2]. The MS pathology transitions into a neurodegenerative disease process associated with insufficient neurotrophic support resulting in irreversible axonal and neuronal loss [3]. This progressive disease commonly affects mobility (i.e. gait function) [4]. Gait impairment in persons with MS (PwMS) has been identified by altered spatio-temporal gait parameters such as slower gait speed, reduced cadence, shorter step length, prolonged stride time and increased double support period [5, 6]. Therefore, accurate assessment of gait characteristics in PwMS is required to examine the severity and progression of gait impairment.

Gait assessment in PwMS has relied on analysis in gait laboratories, with specialized equipment including expensive motion capture systems, pressure sensitive walkways, and force-plates [7]. Clinicians also utilize objective performance based measures (e.g. timed-25 foot walk (T25W)), 6 minute walk test (6MW) [8], and subjective functional ambulation tests (e.g. Performance-Oriented mobility assessment, Dynamic Gait Index)) [8, 9] to assess gait function. However, all of these clinical assessments only provide a ‘snapshot’ of an individual’s walking ability that may not extrapolate to typical gait performance under normal daily conditions.

To overcome these limitations, there has been increasing interest in approaches for objectively examining the walking activity of PwMS in real-world situations [10, 11]. Consequently, body-mounted sensors such as the GT3X (Actigraph, Inc.) have been leveraged to enable long-term ambulatory data collection. These ambulatory monitoring systems may allow for a more granular assessment of gait and a better understanding of MS symptom presentation at the micro (minutes to hours to days) and macro (weeks to months to years) time scales [11].

Activity counts by such wearable activity monitors have been reported to be associated with disability status, and walking impairment in PwMS [12, 13]. There have also been several investigations describing the accuracy of wearable activity monitors for counting steps in populations with gait impairment, including PwMS [14–17]. The GT3X activity monitor has been found to accurately measure step number while walking at moderate and fast speeds in individuals with mild gait impairment [14–16, 18]. However, this sensor significantly underestimates strides at slower walking speeds only among those with severe gait disability [14, 15, 17]. Additionally, the GT3X only counts stride/step numbers and does not record temporal gait parameters (e.g. stride time, swing time) which are important indicators of gait pathology [19].

To measure temporal gait parameters in real life environments over extended durations, one requires light-weight inertial sensors (i.e. accelerometers and gyroscopes) that can be affixed to the body [20]. Indeed, commercially available MTx inertial sensors (Xsens Inc.) have been found to be a valid tool to measure temporal gait parameters during an extended walk (i.e. 6 minute walking test) in PwMS with a range of walking impairment levels [21]. Nevertheless, most inertial sensors are not suitable for monitoring ambulation in daily life as they require relatively cumbersome sensors to be secured to the body with straps and wires.

**Fig 1:**
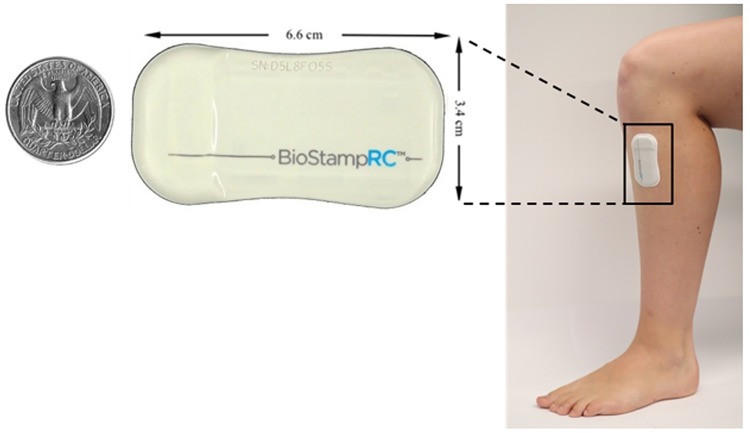
BioStampRC a novel wireless, skin-mounted conformal inertial sensor.

Recently, a wireless, skin-mounted, conformal inertial sensor, BioStampRC (MC10, Inc.) has been developed (Fig 1). The BioStampRC sensors do not constrain or affect natural body motions. The intimate coupling of the BioStampRC sensors to the skin makes this system well suited for testing in a number of settings to assess various aspects of walking. One such assessment is T25W, which has been utilized as a clinical measure of maximal walking speed [8]. Another assessment is 6MW, which is a validated measure of walking endurance [22]. Also, the timed up and go test (TUG) examines dynamic balance and coordination as it includes various aspects of ambulation such as rising from a chair, walking, turning, and sitting [23].

In this study, we applied the BioStampRC system to measure gait characteristics in PwMS during several walking tests and compared the results to commercially available sensors (GT3X and MTx) and visual inspection (manual step count). Importantly, the GT3X and MTx were included as standard measurements because these are commonly used activity monitors and inertial sensors for analyzing ambulation [24, 25]. Also, their accuracy and precision in analyzing gait characteristics has been validated in healthy and neurologic populations [24, 25]. We tested several hypotheses during this study: 1) BioStampRC will distinguish gait characteristics between healthy controls and individuals with MS as well as across walking impairment levels in PwMS; 2) BioStampRC has comparable accuracy and precision to measure gait characteristics of PwMS compared to GT3X and MTx; 3) accuracy of BioStampRC is lower in PwMS with greater walking impairment; and 4) accuracy of the BioStampRC is consistent across the various walking tests.

## 2. Materials and methods

### 2.1. Study participants

45 PwMS and 15 healthy control subjects were recruited in this study. PwMS were recruited from previous subject pools and the local community. The control group was recruited through digital advertisements sent out to the local community. To be included in the MS group, participants were required to have a neurologist-confirmed diagnosis of MS, be able to walk 6 minutes with or without an aid, be at least 18 years old, and have the willingness to wear the inertial sensors used in this study. To be included in the control group, participants were required to have the ability to walk 6 minutes without an aid, have no history of neurological or orthopedic conditions that might influence their balance or mobility, be at least 18 years old, and have the willingness to wear the inertial sensors. PwMS were further divided into groups based on comfortable over-ground walking speed. Specifically, cut points for comfortable walking speed were used to classify subjects into three categories (mild: speed > = 1.1 m/s, moderate: 0.7 < speed < 1.1 m/s, severe: speed < = 0.7 m/s) [26]. A conscious effort to have 15 subjects in each group was made during a recruiting process. The gait impairment level of the participants was estimated during a recruiting process by asking general questions on walking ability and assistive device usage.

### 2.2. Experimental procedures

All procedures were approved by the University of Illinois at Urbana-Champaign institutional review board. Upon arrival at the laboratory, all participants had the experimental procedures explained in detail and were provided an opportunity to ask any questions. When all questions were addressed, participants provided written informed consent. Participants with MS completed several questionnaires concerning their disability including the self-reported expanded disability status scale (EDSS_SR_)[27], the twelve item MS Walking Scale (MSWS) [28] and patient determined disease steps (PDDS) [29] scale. At the end of the ambulation assessment described below, the participants completed a survey focusing on comfort and wearability of each sensor. The participants indicated comfort level utilizing a 1–5 score where 1 was “very comfortable”, 2 was “somewhat comfortable”, 3 was “neither comfortable nor uncomfortable”, 4 was “somewhat uncomfortable”, and 5 was “very uncomfortable”.

#### 2.2.1. Sensors

**Fig 2:**
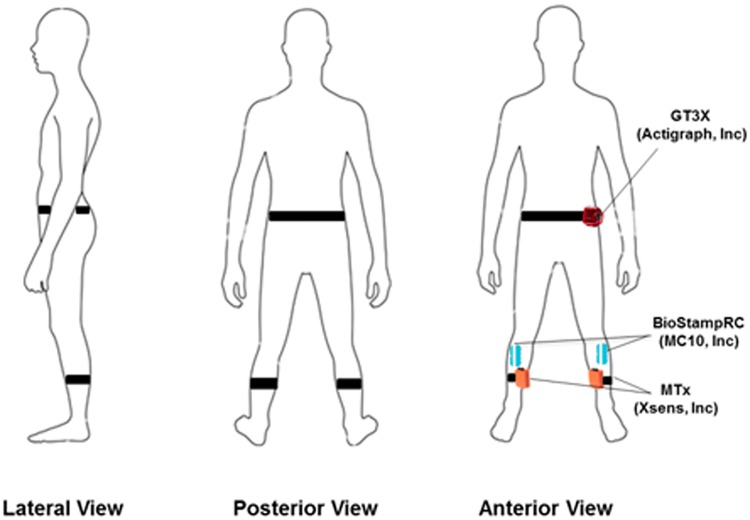
Location of BioStampRC, MTx, and GT3X sensors on the body.

Once participants completed the questionnaires, they were outfitted with three types of wearable motion sensors (Fig 2): 1) BioStampRC devices (MC10, Inc., Lexington, MA), 2) MTx inertial sensors (Xsens Technologies B.V., Netherlands), and 3) tri-axial activity tracker GT3X (Actigraph, Pensacola, FL). The BiostampRC devices were applied to the skin bilaterally on the tibialis anterior in line with tibia tuberosity. The MTx sensors were placed bilaterally on the medial surface of each tibia [18]. The GT3X was placed on the participant’s non-dominant hip with an elastic fabric belt [18]. The locations of each sensor were determined to minimize the skin movement artifacts and interference between the sensors [18, 30].

#### 2.2.2. Gait assessments

After being outfitted with the sensors, the participants underwent a series of mobility and gait assessments. The participants completed a comfortable walking test, Timed 25 feet walk test (T25W), Timed Up and Go test (TUG), and a series of six-minute walking bouts (6MW). All walking tests are validated clinical measures of walking ability and capacity in PwMS [29]. In the comfortable walking test, the subject was instructed to walk for 25 feet over the ground at a self-selected normal, comfortable pace. For the T25W and TUG test, the participants were instructed to walk as quickly but safely as possible [31, 32]. The over-ground comfortable walking test, T25W and TUG tests were completed twice. The time for each trial was manually recorded and was averaged. The use of an assistive device was permitted during the testing.

The 6MW tests were conducted at slow, comfortable and fast walking speeds on a motorized treadmill [10]. The walking speed for comfortable 6MW condition was initially estimated based on the gait speed of the over-ground comfortable walking test. Also prior to a 6MW trial, the participants walked at the estimated comfortable speed on the treadmill and responded whether it would be comfortable to maintain the speed for 6 minutes. Based on their answer, the comfortable walking speed on the treadmill was slightly adjusted. The slow and fast speed settings were determined as 20% above and 20% below the comfortable speed respectively. Those speeds were selected based on previous research that found the maximum safe gait speed of a clinical population was ~20% faster than their comfortable gait speed [33]. Once the speed was determined, the participants were instructed to walk at the designated speed for 6 minutes and were allowed to rest by stopping the treadmill if needed. The participant was allowed to hold on to the rails when walking on the treadmill if necessary for safety purposes; any use of the rails was recorded on a datasheet where necessary.

#### 2.2.3. Data analysis

Step counts were manually measured in the 6MW test and served as a gold standard. The distance covered in 6 minutes was recorded with a calibrated measurement wheel attached directly to the treadmill.

Data collected by BioStampRC and MTx were analyzed to calculate the number of strides as well as temporal gait parameters including stride time, swing time and step time. All data processing was done using custom developed MATLAB code (The MathWorks, Natick, MA, USA).

BioStampRC collected tri-axial acceleration and angular velocity of the shank while MTx recorded tri-axial angle of the shank. Both devices sampled data at 50Hz. A numerical differentiation was performed to the MTx data to obtain angular velocity of the shank recorded by the device. Then the angular velocity of the shank collected by each device was digitally filtered with fourth-order, zero-phase, low-pass Butterworth filter with 10 Hz cutoff frequency [34]. Prior to the calculation of the temporal gait parameters of each trial, the reference frames of the BioStampRC and MTx were aligned using custom developed MATLAB script that employs a singular value decomposition based approach.

**Fig 3:**
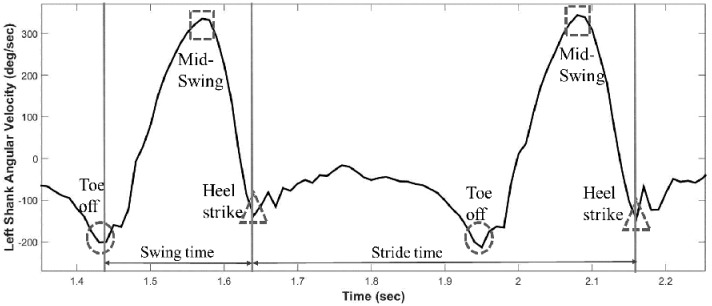
Schematic of shank angular velocity and gait events.

The projection of the shank angular velocity onto the medial-lateral axis was then analyzed to identify the gait events based on a method described previously [20]. This algorithm has been utilized in several investigations analyzing temporal gait parameters in diverse populations including MS [21, 35]. Heel strike point (HS, when the foot first touches the floor) was defined as the time point where the minimum negative peak shank angular velocity occurs immediately following the time point where the positive peak shank angular velocity occurs. This derives from the fact that the shank angular velocity reaches its highest value at the mid-swing phase and at the end of the swing phase the leg is brought to a halt by a heel strike leading to a sharp negative peak of shank angular velocity [36]. Toe off point (TO, when the foot takes off) was defined as the time point where minimum negative peak occurs immediately before the positive peak [20]. Prior to the swing phase, the toe off contributes to progression with a forward push leading to negative peak of shank angular velocity [36] (See Fig 3). Once every HS and TO point was obtained, the temporal gait parameters were calculated using the following equations:

$$Stride\;time\left(i\right)=HS_{Left}\left(i+1\right)-HS_{Left}\left(i\right)\left(1\leq i\leq N-1,\;N:number\;of\;gait\;cycles\right)$$ (1)

$$Swing\;time\left(i\right)=\;TO_{Left}\left(i\right)-HS_{Left}\left(i\right)$$ (2)

$$Step\;time\left(i\right)=HS_{Left}\left(i\right)-HS_{Right}\left(i\right)$$ (3)

For Actigraph GT3X data, the sensor recorded tri-axis acceleration at 30Hz in 6MW trials. The data was processed to count step numbers with ActiLife 6 software (Actigraph, Pensacola, FL) in one second epoch intervals (i.e. sampling window). Following the manufacturer’s guideline [17, 37], a low frequency extension filter was used as it increases the sensitivity for detecting low-frequency accelerations (i.e. slow walking).

GT3X was included as standard measurements for step count since it is the most commonly used activity monitor for analyzing ambulation in healthy and diseased populations, including MS [24]. MTx served as a reference standard for assessing temporal gait parameters as its accuracy to measure these has been validated [25, 34].

#### 2.2.4. Statistical analysis

Accuracy (mean of error) and precision (variance of error) were reported based on absolute and relative metrics. Absolute accuracy was defined as a difference between gait parameters recorded by devices and those recorded by the reference standards. The reference standard for a step number was visual inspection (manual count) while the reference standard for temporal parameters was those derived from MTx [34]. The absolute accuracy was presented by the median and the inter quartile range (IQR) for step numbers and by mean and 95% confidence interval (CI) for temporal gait parameters. Relative accuracy was based on percentage error $\text{percentage error }(=\frac{\text{tested equipment}-\text{standard equipment}}{\text{standard equipment}}\times 100)$. Absolute precision (variance of error) was based on the standard deviation of the absolute errors recorded per device, whereas relative precision involved standard deviation of the relative errors per device. Also, Bland-Altman plots were generated for visually examining systematic patterns of error in estimation of temporal gait parameter.

Difference of gait parameters between the groups and the devices were assessed with two-way (group × device) ANOVA analysis. Also, differences of accuracy of gait parameters relative to the device, group and tests were examined with three-way (device × group × test) ANOVA analysis. When appropriate, Bonferroni analysis was used for post-hoc analysis. Group differences of parametric data of demographics were assessed using ANOVA analysis. For non-parametric data, Kruskal-Wallis test was conducted to examine group differences followed by Mann-Whitney U test for post-hoc analysis. All analysis used two-sided tests, and p-values equal to or less than 0.05 were considered statistically significant. The analysis was performed with IBM SPSS statistics for Windows (version 22; IBM SPSS Inc., Armonk, NY, USA).

As there were no differences in the results of the accuracy analysis between left and right sides, we only reported left side results for brevity. The data for the right side are reported separately in the supplementary data section (S1 and S2 Tables).

## 3. Results

### 3.1. Demographics

The final sample included 60 adults divided equally into 4 groups based on presence of MS and gait speed: healthy controls (n = 15), mild walking impairment MS (n = 15), moderate walking impairment MS (n = 15) and severe walking impairment MS (n = 15). The demographics and clinical characteristics of the groups are provided in Table 1. Per design, over-ground comfortable walking speeds were not different between the control and mild MS group (p>0.05) while it was significantly reduced with increasing impairment level in PwMS (p’s<0.05). There was no group difference in age, height, weight and MS duration [F(3,59) = 1.39, p = 0.26; F(3,59) = 1.51, p = 0.22; F(3,59) = 2.09, p = 0.14; F(3,59) = 0.91, p = 0.44, respectively]. EDSS, MSWS and PDSS were significantly different among the MS groups [χ^2^(2) = 27.9,p<0.01; χ^2^ (2) = 20.4,p<0.01; χ^2^ (2) = 24.1,p<0.01, respectively]. Post-hoc analysis showed that MSWS and PDSS score were significantly different between each group (p’s<0.05) while there was no significant difference in EDSS between the moderate and severe group [U = 81, *Z* = -1.4, p = 0.10]. Overall, 23% of the participants used a cane and 7% used a walker during over-ground trials. A total of eight subjects wore an ankle-foot orthosis (AFO) during the test (moderate: 3 subjects, severe: 5 subjects). Also, 53% of the participants held on to the support rails during 6MW test (control: none, mild MS: 4 subjects, moderate MS: 12 subjects, severe MS: 15 subjects).

**Table 1:** Demographics and clinical characteristics.

	Control	Mild MS	Moderate MS	Severe MS
N	15 (10F/ 5M)	15 (12F/ 3M)	15 (10F/ 5M)	15 (10F/ 5M)
Age (yrs)	57.9±12.9	53.7±12.3	59.7±8.3	61.1±8.3
Height (cm)	169.0±6.7	165.9±7.7	171.7±8.6	168.0±7.0
Weight (kg)	77.4±15.0	75.1±15.9	81.5±16.3	86.4±28.9
MS duration (yrs)	--	14±8	18±6	21±10
Assistive device (none/cane/ walker)	15/0/0	15/0/0	7/7/1	4/8/3
EDSS_SR_ (Median(IQR))	--	1.5 (0–2.5)	6.0 (3.5–6)	6.0 (5.5–6)
MSWS (Median(IQR))	--	17 (14–20)	43 (26–49)	48 (43–55)
PDDS (Median(IQR))	--	0 (0–1)	4(3–5)	5 (4–5)
Over-ground comfortable walking speed (m/s)	1.28±0.18	1.28±0.17	0.86±0.10	0.60±0.12

### 3.2. Performance of gait assessments

Table 2 demonstrates the performance outcomes of the clinical gait assessments by cohort. Overall, there was group effect in all of the tests [T25W: F(3,59) = 22.6, p<0.01; TUG: F(3,59) = 30.2, p<0.01; 6MW comfortable: F(3,59) = 33.3, p<0.01; 6MW slow: F(3,59) = 35.8, p<0.01; 6MW fast: F(3,58) = 36.9, p<0.01].

**Table 2:** Performance of clinical gait assessments as a function of groups.

Test	Control	Mild MS	Moderate MS	Severe MS
T25W (sec)	4.22±0.54	5.10±0.80	6.81±1.04*	10.6±4.41*†δ
TUG (sec)	5.86±1.01	7.28±1.19	11.0±3.24*†	16.5±5.72*†δ
6MW_Comfortable (m)	424±127	443±102	256±67*†	135±54*†δ
6MW_Slow (m)	339±100	354±83	206±68*†	105±43*†δ
6MW_Fast (m)	504±139	536±115	303±101*†	168±58*†δ

Note: significantly (p<0.05) different from

*the controls;

^†^the mild MS;

^δ^ the moderate MS

The post-hoc analysis revealed that no difference in gait performance between the control and the mild MS group in all of the tests (p’s>0.05). There was significant reduction in gait performance as a function of severity level in the MS groups (p’s<0.01). One participant in the severe MS group chose not to complete 6MW at fast speed condition. The subject was removed from the pool for the fast 6MW.

### 3.3. Gait parameters recorded by BioStampRC

Table 3 displayed gait parameters recorded by BioStampRC as a function of group. There was no significant difference between control and mild MS groups in any gait parameters (p’s>0.05). Significantly increased stride time and step time and decreased step number were observed with increasing impairment level in the MS cohort (p’s<0.01). Swing time of the moderate and severe group was not significantly different in 6MW comfortable and slow speed tests (p’s>0.05). Also swing time of the control and moderate group was not significantly different in over-ground comfortable walking and 6MW comfortable speed tests (p’s>0.05). There was neither significant device (MTx vs BioStamp) effect nor group*device effect in all of the tests (p>0.05).

**Table 3:** Gait parameters recorded by BioStampRC as a function of group and speed.

Test	Gait parameter	Control	Mild MS	Moderate MS	Severe MS
Over-ground comfortable walking	Stride time (ms)	1083±111	1035±64	1239±101*†	1594±419*†δ
	Swing time (ms)	445±40	437±29	467±40†	521±87*†δ
	Step time (ms)	543±53	522±36	619±51*†	800±208*†δ
T25W	Stride time (ms)	874±108	915±50	1079±121*†	1369±368*†δ
	Swing time (ms)	383±43	399±30	430±34*†	478±85*†δ
	Step time (ms)	422±52	463±26	542±60*†	687±18*†δ
TUG	Stride time (ms)	856±122	929±85	1082±172*†	1468±408*†δ
	Swing time (ms)	378±51	395±41	427±43*†	481±92*†δ
	Step time (ms)	429±58	471±43	549±93*†	744±211*†δ
6MW_Comfortable	Step number	636±184	692±60	484±150*†	392±94*†
	Stride time (ms)	1076±92	1047±96	1385±193*†	1857±40*†δ
	Swing time (ms)	461±33	447±29	498±57†	534±87*†
	Step time (ms)	538±46	523±46	693±97*†	929±202*†δ
6MW_Slow	Step number	562±168	614±68	466±74*†	340±92*†δ
	Stride time (ms)	1214±128	1184±141	1587±285*†	2233±600*†δ
	Swing time (ms)	486±38	482±41	543±76*†	574±101*†
	Step time (ms)	607±64	592±705	793±142*†	1047±414*†δ
6MW_Fast	Step number	680±204	736±78	548±110*†	380±150*†δ
	Stride time (ms)	1009±109	987±103	1342±267*†	1668±596*†δ
	Swing time (ms)	440±34	425±41	495±60*†	546±95*† δ
	Step time (ms)	505±54	494±51	671±133*†	833±297*†δ

Note: Significantly (p<0.05) difference from

*the controls;

^†^the mild MS group;

^δ^ the moderate MS group

### 3.4. Accuracy and precision of step number count

Table 4 presents the data for accuracy and precision of step number count per device when comparing to the reference standard (manual count). The absolute error of the BioStampRC and MTx both had median values of two steps (IQR: 2—6steps) when compared to the reference standard. GT3X had median absolute error of 10 steps (IQR: 3–38 steps).

**Table 4:** Accuracy and precision of stride/step number count per device.

Test	Device	Gait parameter	Absolute accuracy		Relative accuracy				Precision	
			Medianerror	IQR	% error	5%<N	10%<N	15%<N	ABS	REL
6MW_Comfortable	BioStampRC	Step N diff	2	2–4	0.8%	1	0	0	3	1.2%
	MTx	Step N diff	2	2–4	0.9%	0	0	0	3	1.2%
	GT3X	Step N diff	10	3–48	10.1%	19	14	11	78	17.0%
6MW_Slow	BioStampRC	Step N diff	2	2–6	1.0%	1	0	0	2	1.3%
	MTx	Step N diff	2	2–4	1.0%	1	0	0	2	1.2%
	GT3X	Step N diff	13	2–56	9.4%	23	17	10	53	14.0%
6MW_Fast	BioStampRC	Step N diff	4	2–6	0.9%	1	0	0	3	1.2%
	MTx	Step N diff	4	2–6	0.9%	1	0	0	2	1.0%
	GT3X	Step N diff	6	2–20	6.1%	13	8	7	57	12.7%

Note: Total N = 60 for each test, ABS = absolute, REL = relative

The mean relative accuracy ± precision was 0.9±1.2% for BioStampRC, 0.9±1.1% for MTx and 8.1±14.2% for GT3X. Only three cases (2%) of step number data from BioStampRC and two cases (2%) from MTx demonstrated ≥ 5% relative error. On the contrary, 55 cases (31%) of the GT3X data showed ≥ 5% relative error in the step number record.

There was significant difference in relative error between the devices [F(2,348) = 163.8, p<0.01]. Post-hoc analysis revealed that absolute and relative error of GT3X was significantly greater than that of BioStampRC and MTx (p<0.01). There was no significant difference of absolute and relative error between BioStampRC and MTx (p = 1.00).

Subsequent analysis revealed that the GT3X had significantly greater error in step number in the severe group (24±19% error) than that in the other groups (control: 1±2% error; mild MS: 1±2% error; moderate MS: 5±8% error) [F(3,174) = 32.7, p<0.01]. There was no significant group difference in step number error in BioStampRC and MTx (p’s>0.05).

There was no significant difference in the error between the tasks with all of the devices [BioStampRC: F(2, 174,) = 0.131, p = 0.88; MTx: F(2,174) = 0.64, p = 0.94; GT3X: F(2,174) = 1.20, p = 0.30]. Also there was no interaction effect of group, device and task (p’s>0.05).

### 3.5. Accuracy and precision of temporal gait parameters recorded by BioStampRC

**Fig 4:**
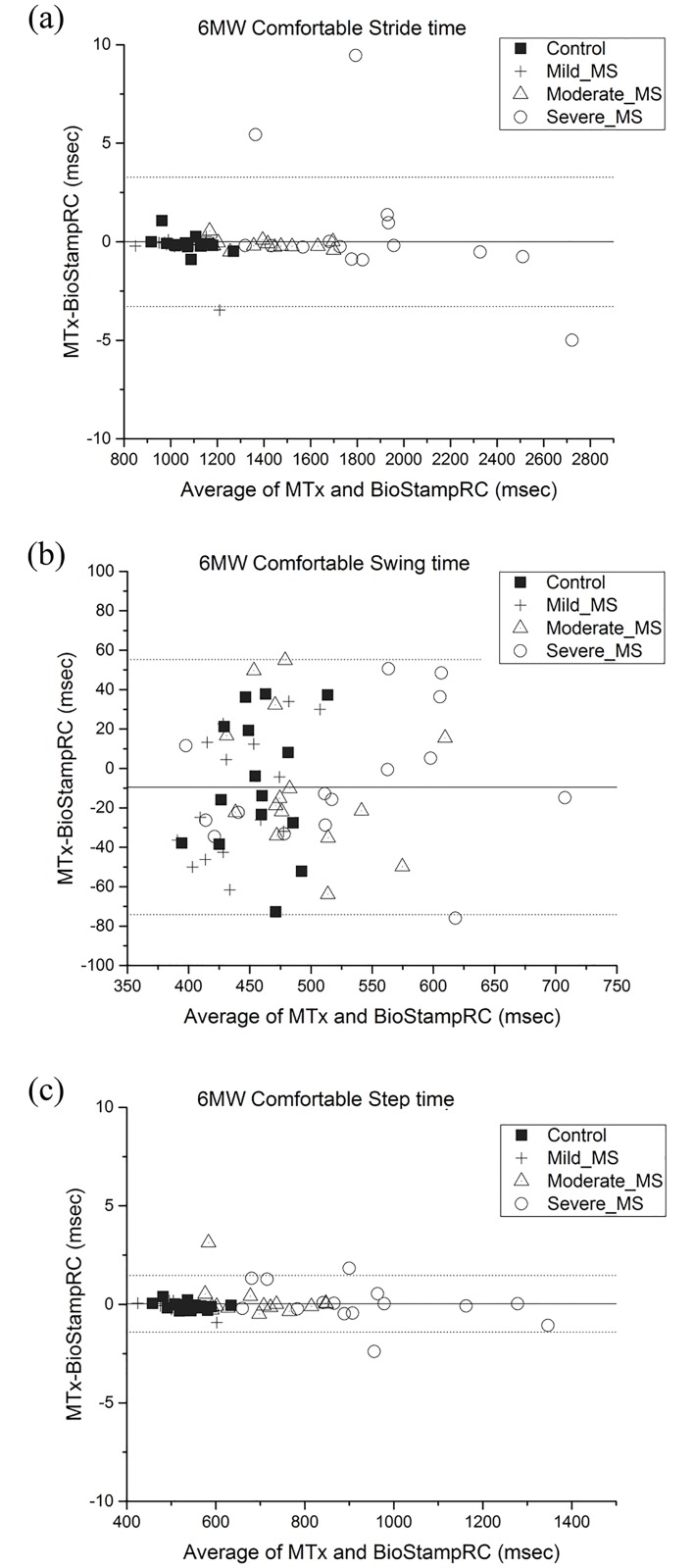
Bland-Altman plot of (a) Stride time (b) Swing time (c) Step time of BioStampRC compared with that of reference system (MTx). Limits of agreement are specified as average difference (solid line) and ±1.96 standard deviation of the error (dotted line).

Table 5 presents accuracy and precision of temporal gait parameters collected by BioStampRC when comparing to the reference standard (MTx). Fig 4. illustrates Bland-Altman plot of temporal gait parameters measured by BioStampRC and reference system (MTx) for 6MW comfortable speed. The graphical comparisons for other assessments are reported in the supplementary data section (S1 Fig).

**Table 5:** Accuracy and precision of temporal gait parameters measured by BioStampRC.

Test	Gait parameter	Absolute accuracy		Relative accuracy				Precision	
		Meanerror	95% CI	Mean error	5%<N	10%<N	15%<N	ABS	REL
Over-ground comfortable walking	Stride time diff (ms)	8.9	5.2–12.6	0.6%	0	0	0	12.0	0.9%
	Swing time diff (ms)	20.6	17.3–23.9	4.4%	18	5	1	17.8	3.6%
	Step time diff (ms)	7.1	4.4–9.7	1.2%	1	0	0	9.1	1.5%
T25W	Stride time diff (ms)	8.3	6.0–10.5	0.8%	0	0	0	11.8	1.3%
	Swing time diff (ms)	21	17.8–24.2	5.0%	19	5	1	16.9	4.1%
	Step time diff (ms)	7.4	5.7–9.2	1.4%	1	0	0	9.4	1.4%
TUG	Stride time diff (ms)	12.6	9.7–15.5	1.2%	1	0	0	14.9	1.2%
	Swing time diff (ms)	26.9	23.2–30.6	6.5%	32	18	7	23.2	5.0%
	Step time diff (ms)	14.1	11.4–16.8	2.6%	7	0	0	12.5	2.1%
6MW_Comfortable	Stride time diff (ms)	0.7	0.3–1.1	0.0%	0	0	0	1.5	0.1%
	Swing time diff (ms)	29.8	24.7–35.0	6.3%	31	9	1	19.0	3.9%
	Step time diff (ms)	0.4	0.2–0.6	0.0%	0	0	0	0.6	0.1%
6MW_Slow	Stride time diff (ms)	1.6	0.4–2.8	0.0%	0	0	0	4.5	0.2%
	Swing time diff (ms)	27.5	22.3–32.8	5.3%	24	6	1	19.7	3.7%
	Step time diff (ms)	0.9	0.4–1.3	0.0%	0	0	0	1.8	0.2%
6MW_Fast	Stride time diff (ms)	1.2	0.4–2.1	0.1%	0	0	0	3.2	0.2%
	Swing time diff (ms)	29.7	24.3–35.0	6.1%	30	9	0	20.0	3.8%
	Step time diff (ms)	0.6	0.3–0.9	0.1%	0	0	0	1.1	0.1%

Note: Total N = 60 for each test, ABS = absolute, REL = relative

The absolute accuracy±precision of stride time and step time was 6.3±8.7ms and 5.8±7.1ms, respectively, when compared to the reference standard. The absolute accuracy±precision of swing time was the worst (24.6±18.9ms) among the temporal gait parameters.

The mean relative absolute±precision error was 0.5±0.9%, 5.4±4.1% and 1.0±1.5% for stride time, swing time and step time, respectively. Only one case of stride time data (1%) and 9 cases of step time data (2%) derived from the BioStampRC had ≥ 5% relative error. Swing time data had the greatest frequency of data with ≥ 5% relative error (154 cases, 43% of data) with a maximum of 20% relative error.

There was a significant group difference in absolute and relative error in stride time and step time (p’s<0.01). Post-hoc analysis revealed that there was significantly greater error in stride time and step time of the severe MS group (stride time error: 10.6 ms, 0.6% error; step time error: 6.5ms, 1.3% error) compared to that of the other groups (p’s<0.01). There was no significant difference in swing time accuracy between the groups in all gait assessments (p’s>0.05).

Also, there was significant difference in absolute and relative error in stride time and step time between the clinical assessments (p’s<0.01). Post-hoc analysis showed that there was significantly greater error in stride time and step time during the TUG than that of the other assessments (stride time: p = 0.05; step time: p<0.01). Also, there was significantly less error in stride time and step time (p’s <0.01) during 6MW than during the other assessments. There was no difference in the error between 6MW tests with different speed conditions (p’s>0.05). There was no significant difference in swing time error between the gait assessments (p’s>0.05).

### 3.6. Sensor wearability

All of the participants reported that they felt comfortable when wearing BioStampRC (Median: 1, IQR: 1–1) and GT3X (Median: 1, IQR: 1–1) to the extent they were not aware of the sensors during the assessment. However, 32% of the participants reported MTx was uncomfortable (Median: 2, IQR: 1–3) due to the cumbersome sensor hub around the waist, the wires affecting natural movement, and the straps compressing their shins during walking.

## 4. Discussion

The primary objective of this study was to examine the accuracy and precision of a novel wearable device, the BioStampRC, as a measure of gait in PwMS with a wide range of gait function. Our results show that the BioStampRC has comparable or improved accuracy in measuring step number and temporal gait parameters compared to commercially available research grade inertial sensors and accelerometers. Additionally, the BioStampRC detected altered gait characteristics in PwMS (i.e., elongated stride time, swing time, and step time). Taken together, these results demonstrate that BioStampRC has sufficient accuracy and precision for gait measurement in clinical research involving PwMS in controlled settings.

### Step number was the most inaccurate in GT3X

Both BioStampRC and MTx demonstrated highly accurate and precise measurements of step number (0.6% underestimated step number) across all groups and all speed conditions of 6MW. GT3X showed the highest error in counting steps (8% underestimated step number).

The trunk and hip, where the GT3X is located, experience reduced accelerations in the medial-lateral and vertical directions during slow walking especially when an assistive device or support is used [38]. The algorithm present in the ActiLife software calculates steps based on vertical accelerometer data after filtering out the baseline noise level [37]. Following the manufacturer’s guidelines, GT3X data were processed with a unique filtering mode, the low frequency extension filter to improve sensitivity to detect steps in lower amplitude movements [17, 37]. However, despite usage of the unique filtering system, GT3X still significantly underestimated the step number of the severe MS group. This observation corresponds to a previous study that found overall GT3X had 5% error in step counts for PwMS and it had the greatest error for the PwMS with severe walking disability (13% error) [14]. Additionally, it should also be noted that all of the participants in the severe MS group spontaneously held on to the rails on the treadmill during 6MW. Previous research found that GT3X had increased error in step count for participants walking with an aid (17% underestimated error) [17].

It is possible that the recommended algorithm might not be sensitive enough to detect steps with minimized vertical acceleration of the hip due to characteristics of gait impairment and assistive device use. Considering that gait impairment is a common symptom of MS and nearly half of PwMS use an assistive device during walking [4], relying solely on vertical acceleration of the hip is problematic and may lead to significant measurement error. It has been suggested that step count accuracy was best when activity monitors were located distally such as on the ankle [17, 39]; however, despite this fact, the GT3X is frequently worn at the waist to minimize its interference with daily life activities [17]. Thus, both sensor location and the algorithm for assessing impaired gait may contribute to the errors in the GT3X results.

### Swing time was the least accurate and precise

In terms of assessing temporal gait parameters, BioStampRC demonstrated highly accurate and precise calculations of stride time (6±9ms, 0.5±0.9% error on average) and step time (6±7ms, 1.0% error on average) whereas there was less accuracy with the swing time calculation (25ms, 6.1% error on average). The algorithm utilized to determine temporal gait parameters was based on the work of Aminian, et al. They also reported that stance time calculations, which is the mathematical inverse of swing time, were less accurate (23ms error) compared to stride time (8ms error) [20]. Additionally, inaccuracy in swing time has been consistently observed in studies that have utilized the same algorithm for measuring gait events with gyroscope sensors mounted on the lower limb (35-55ms error) [34, 40, 41]. However, the origin of the swing time error is controversial as some investigations reported the error at heel strike detection [20, 34], whilst others reported the bias at toe off detection [40]. The discrepancy might be due to the fact that the investigations used pressure sensor switches as a reference standard and the choice of sensor threshold critically influences the detection of gait events [40]. Therefore, refinements in the algorithm to enhance accuracy of estimating swing time are warranted. It should be noted that both step time and stride time rely solely on heel-strike events (Eqs 1 & 3), while swing time also uses toe-off events (Eq 2). Therefore, improving systematic heel-strike and toe-off detection may improve swing-time algorithm performance. Algorithm refinements as well as incorporating additional data sources such as three dimensional accelerometer data may improve accuracy in detecting swing phase. Importantly, the swing time error, which demonstrated the greatest error among the gait parameters, was not different between the groups. This suggests that the origin of the swing time error is not relevant to gait impairment level of the participants.

### Accuracy of gait parameter measurement was the least in TUG and the greatest in 6MW

There are a few possible reasons that error in stride time and step time was greatest during the TUG test. The increased error might be due to inclusion of diverse movement during the test such as turning, sitting, standing, initiation and termination of gait [42]. The accuracy of the gait detection algorithms based on the inertial sensors was only investigated in continuous straight-line walking [20, 34]. Therefore, the various movements included in TUG might increase inaccuracy of calculation of gait parameters detected by the BioStampRC and MTx. This observation is important as the dynamic movements required in the TUG test have been suggested to have increased relevance in community ambulation [42]. In order to utilize the inertial sensors to monitor daily life walking, the algorithm for detecting gait events may need to be improved so that it can be applied to diverse ambulation tasks. Additionally, the 6MW had better accuracy and precision compared to the short distance walking tests. The greater number of steps during 6MW might eliminate influence of outliers and increase the accuracy and precision of the measurement. This observation is important considering that the BioStampRC sensor has potential to provide long-term gait monitoring.

### The severe MS group had greater stride and step time error

The greatest error in stride time and step time occurred in the severe MS group. The gait impairment characteristics such as slowed angular motion of the shank and a dragged foot might lead to the increased inaccuracy of detecting gait events using the algorithm based on shank angular velocity [43]. However, it should be noted that despite the increased error, the stride time and step time still demonstrated minimal absolute and relative error in the severe group (stride time error:10.6ms, 0.6% error; step time error: 6.5ms, 1.3% error).

### BioStampRC could monitor gait impairment of MS.

BioStampRC sensors detected differences in step time and stride time by disability levels in PwMS, while swing time was less distinctive between the groups. The current observation was consistent with previous researches that reported significant prolongation in step time and stride time, but not in swing time in PwMS [6, 21]. Also, corresponding to previous research [6, 19], there was no difference in the gait parameters between the control and mild MS group whereas elongated temporal gait parameters were observed as severity of gait impairment increased in PwMS. The trend was also observed in the data derived from the standard reference MTx [21, 44]. Therefore, BioStampRC might be useful in identifying gait pathology in PwMS as well as in evaluating the progression of gait disability in PwMS.

### Strengths and limitations of the investigation

The current study included a relatively large sample of persons with varying MS disability as well as healthy controls. Furthermore, we studied four types of clinical gait assessments that have been validated to examine gait function in PwMS [8]. Additionally, three different speed conditions were included in 6MW.

Despite the strengths of the paper, it is not without limitations. A main limitation of the study is that 6MW trials were conducted on a motorized treadmill. It is established that treadmill walking is distinct from over ground walking. The use of treadmill and usage of the hand rail have been associated with altered gait characteristics such as increased cadence and reduced knee angle compared to over-ground walking condition [45, 46]. Additionally, a few studies have suggested that treadmill may artificially reduce the natural variability of gait patterns (i.e. stride-to-stride fluctuations during walking) [46]. The alternation of the gait variability has been reported to be a unique marker of gait impairment in PwMS [47]. Therefore, future research is necessary to confirm the accuracy of the novel sensor and its applications in PwMS during over-ground walking that mimics real-world walking.

The treadmill was utilized in an effort to maintain gait speed within a given trial. The well-controlled speed constraints permitted more explicit comparison of the temporal gait parameters between the speed conditions and devices. The current investigation serves as a starting point in the assessment of accuracy of the novel sensor in a controlled manner. In order to minimize impact of the treadmill condition on natural gait characteristics, we established the walking speed based on over-ground comfortable walking speed. Furthermore, the distance walked during the 6MW in the current study were similar to previous investigations of the over-ground 6MW test [21, 48]. Even so, treadmill walking might still be a novel task to some participants and therefore not an ideal representation of real-life walking.

Also, while the inertial sensors provide tri-axial angular velocity as well as acceleration data, the study only utilized angular velocity data to analyze temporal gait parameters. The use of the angular velocity derived from a gyroscope signal is advantageous since unlike accelerometers, gyroscopes are less sensitive to the influence of gravity and therefore the signal is less influenced by accurate sensor placement [49]. However, it has been reported that angular velocity signal recorded by body-worn gyroscopes can be affected by noise and artifact [49]. A few studies have developed algorithms to analyze the acceleration data to examine gait characteristic of healthy and clinical populations [50]. Therefore, it is promising to examine whether fusing acceleration and angular velocity data improves the accuracy of computed temporal gait parameters.

Further, the different devices used were not attached in the same location on the body. While this helped to minimize interference between devices, there might be some error due to the different attachment locations. To overcome this, an additional computation was conducted to match the orientation between BioStampRC and MTx. Indeed, the current study observed improvement in accuracy of temporal parameters after the adjustment. Also, the study did not include gold-standard for estimating the true outcomes for temporal gait parameters. However, previous studies have been found that the accuracy of MTx in measuring gait is valid when compared to the gold standards (motion capture system, pressure sensor switches) [25, 34]. Lastly, gait speed was utilized to distinguish gait impairment levels of PwMS since it has been reported as an objective spatiotemporal measure of gait impairment [51].

A major benefit of these novel skin-mounted, conformal inertial sensors is their broader applicability to monitoring gait in free-living environment. In the present study, all of the participants reported that they felt comfortable when wearing BioStampRC sensors to the extent they were unaware of the sensors during gait assessments. An essential next step will be to confirm their validity to measure gait characteristics in real-life ambulation.

## 5. Conclusion

We demonstrate that the BioStampRC provides sufficient data to enable a highly accurate measurement of step count and temporal gait parameters in diverse clinical gait tests and speed conditions across varying disability in PwMS. Accuracy was the lowest in swing time such that the error increased up to 5% on average. Improving the algorithm used to detect gait events in order to enhance the accuracy of swing time detection is suggested. BioStampRC was also able to detect differences in gait characteristics by disability progression in PwMS. This ambulatory monitoring system has the potential to provide a more granular assessment of gait and a better understanding of MS related changes in walking symptoms outside of the clinic. Such real-life continuous gait monitoring might overcome the limitations inherent in taking ‘snapshots’ of patient gait state in clinical or laboratory settings. Thus, long-term gait monitoring systems might exhibit greater sensitivity in detecting onset and changes in drug therapy efficacy. A future study confirming the validity of the sensor’s ability to measure gait activities in everyday life is warranted.

## Supporting information

S1 Table Right side gait parameters recorded by BioStampRC as a function of group and speed. (DOCX)

S2 Table Right side accuracy and precision of temporal gait parameters measured by BioStampRC. (DOCX)

S1 Fig Bland-Altman plot of gait parameters measured by BioStampRC and MTx. (DOCX)

S1 Dataset Gait performance. (XLSX)

S2 Dataset Step count. (XLSX)

S3 Dataset Temporal gait parameters. (XLSX)
