# Supplementary material for: Monitoring gait in multiple sclerosis with novel wearable motion sensors
Source: PLoS One. 2017 Feb 8;12(2):e0171346. doi: 10.1371/journal.pone.0171346 (PMC5298289; doi:10.1371/journal.pone.0171346)
Supplement: S1 Table — (DOCX) [file pone.0171346.s001.docx]

S1 Table. Right side gait parameters recorded by BioStampRC as a function of group and speed

| **Test** | **Gait parameter** | **Control** | **Mild MS** | **Moderate MS** | **Severe MS** |
| --- | --- | --- | --- | --- | --- |
| Overground comfortable walking | Stride time (ms) | 1084±111 | 1042±69 | 1238±101*† | 1585±394*†δ |
|  | Swing time (ms) | 448±41 | 444±38 | 492±56*† | 547±89*†δ |
| T25W | Stride time (ms) | 881±114 | 923±55 | 1078±126*† | 1371±369*†δ |
|  | Swing time (ms) | 387±43 | 400±31 | 454±55*† | 510±75*† |
| TUG | Stride time (ms) | 860±122 | 939±87 | 1090±177*† | 1477±422*†δ |
|  | Swing time (ms) | 402±50 | 427±40 | 469±71* | 530±83*†δ |
| 6MW_Comfortable | Stride time (ms) | 1076±92 | 1046±95 | 1385±193*† | 1855±403*†δ |
|  | Swing time (ms) | 441±34 | 445±30 | 514±61*† | 544±99*† |
| 6MW_Slow | Stride time (ms) | 1214±128 | 1184±141 | 1587±283*† | 2246±621*†δ |
|  | Swing time (ms) | 485±41 | 471±72 | 562±74*† | 572±113*† |
| 6MW_Fast | Stride time (ms) | 1009±109 | 987±103 | 1344±268*† | 1786±392*†δ |
|  | Swing time (ms) | 435±30 | 431±32 | 517±68*† | 545±98*† |
