# Supplementary material for: Monitoring gait in multiple sclerosis with novel wearable motion sensors
Source: PLoS One. 2017 Feb 8;12(2):e0171346. doi: 10.1371/journal.pone.0171346 (PMC5298289; doi:10.1371/journal.pone.0171346)
Supplement: S2 Table — (DOCX) [file pone.0171346.s002.docx]

S2 Table. Right side accuracy and precision of temporal gait parameters measured by BioStampRC

| **Test** | **Gait parameter** | **Absolute accuracy** | | **Relative accuracy** | | | | **Precision** | |
| --- | --- | --- | --- | --- | --- | --- | --- | --- | --- |
|  |  | **Mean** | **95% CI** | **Mean error** | **5%<N** | **10%<N** | **15%<N** | **ABS** | **REL** |
| Overground comfortable walking | Stride time diff (ms) | 10.7 | 7.7-13.6 | 0.9% | 1 | 0 | 0 | 12.2 | 0.9% |
|  | Swing time diff (ms) | 28.4 | 24.4-32.4 | 6.1% | 26 | 10 | 3 | 21.4 | 5.1% |
| T25W | Stride time diff (ms) | 12.4 | 6.4-18.4 | 1.2% | 1 | 0 | 0 | 10.8 | 1.0% |
|  | Swing time diff (ms) | 27.8 | 23.6-31.9 | 6.7% | 29 | 10 | 4 | 22.8 | 6.5% |
| TUG | Stride time diff (ms) | 13.8 | 10.9-16.8 | 1.4% | 2 | 0 | 0 | 10.5 | 1.1% |
|  | Swing time diff (ms) | 37.1 | 32.0-42.2 | 9.1% | 35 | 21 | 10 | 29 | 7.4% |
| 6MW_Comfortable | Stride time diff (ms) | 1.7 | 0-0.3 | 1.1% | 0 | 0 | 0 | 6.5 | 0.4% |
|  | Swing time diff (ms) | 27.8 | 21.4-34.3 | 6.2% | 23 | 11 | 3 | 24.6 | 6.1% |
| 6MW_Slow | Stride time diff (ms) | 0.9 | 0.2-1.6 | 0.5% | 0 | 0 | 0 | 2.8 | 0.1% |
|  | Swing time diff (ms) | 32.4 | 24.7-40.1 | 6.9% | 25 | 12 | 7 | 29.7 | 7.3% |
| 6MW_Fast | Stride time diff (ms) | 1.2 | 0.1-2.2 | 0.8% | 0 | 0 | 0 | 4.1 | 0.2% |
|  | Swing time diff (ms) | 26.9 | 21.4-32.5 | 5.9% | 24 | 10 | 3 | 21.4 | 4.9% |
