# Supplementary figures and images for: Monitoring gait in multiple sclerosis with novel wearable motion sensors
Source: PLoS One. 2017 Feb 8;12(2):e0171346. doi: 10.1371/journal.pone.0171346 (PMC5298289; doi:10.1371/journal.pone.0171346)

S3 Figure. Bland-Altman plot of gait parameters measured by BioStamp and MTx.


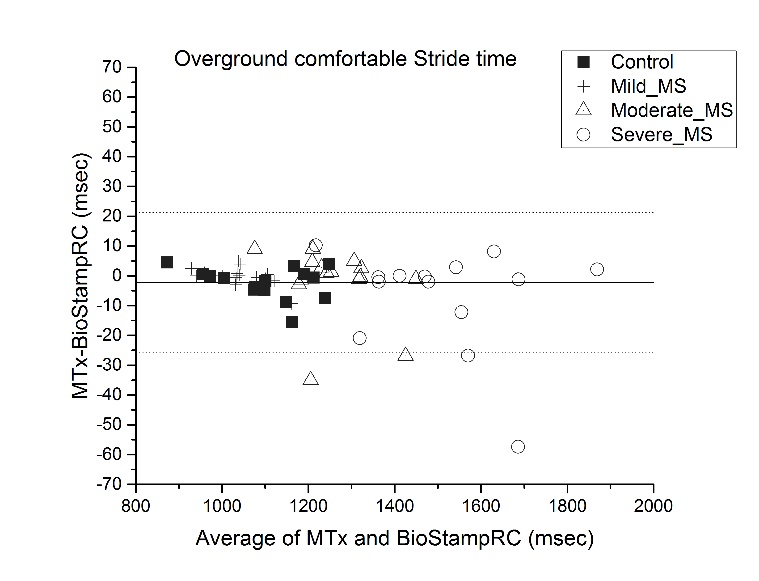

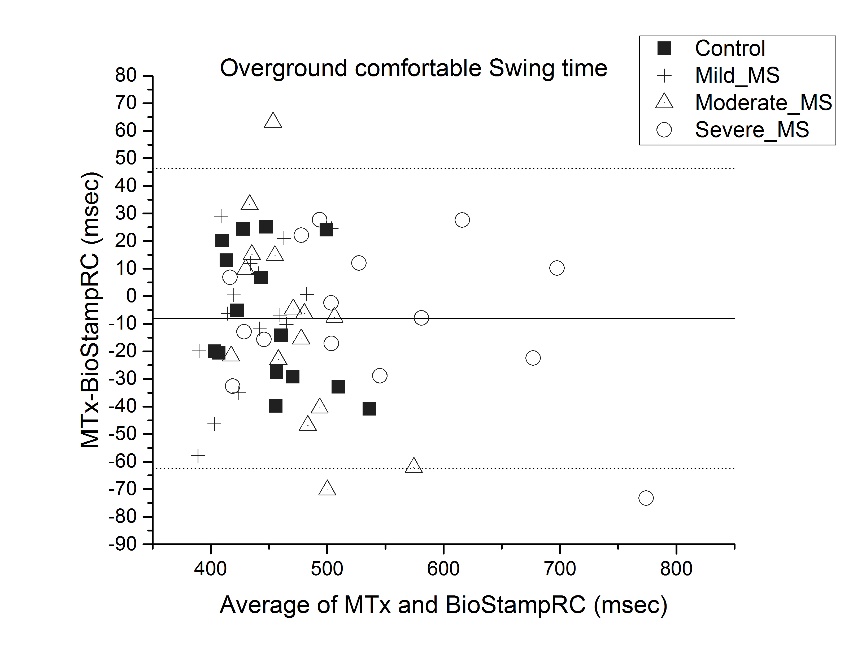

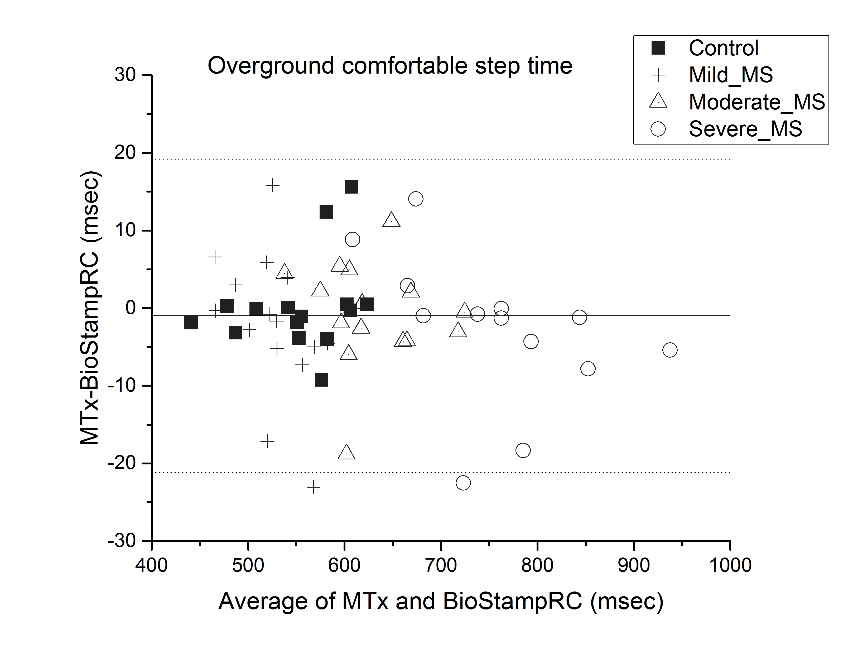


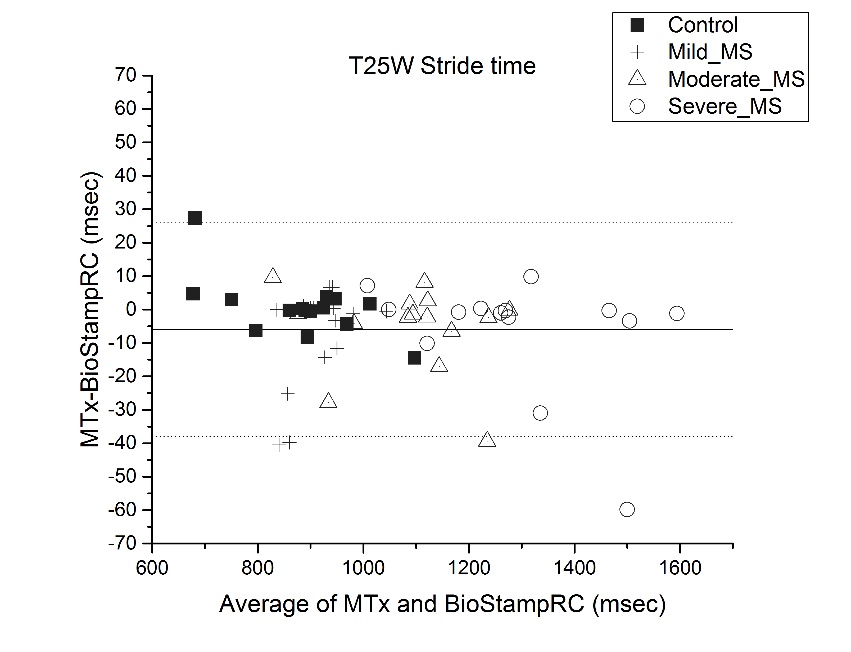


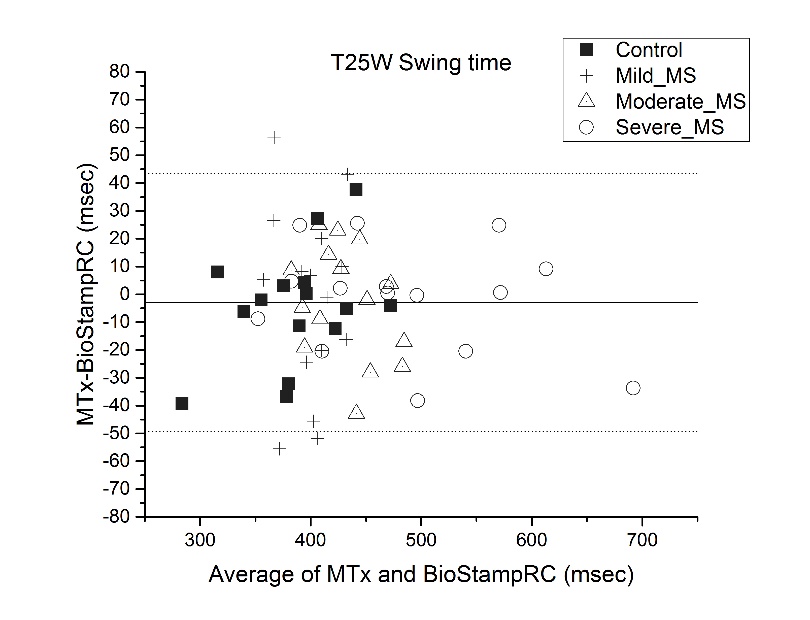


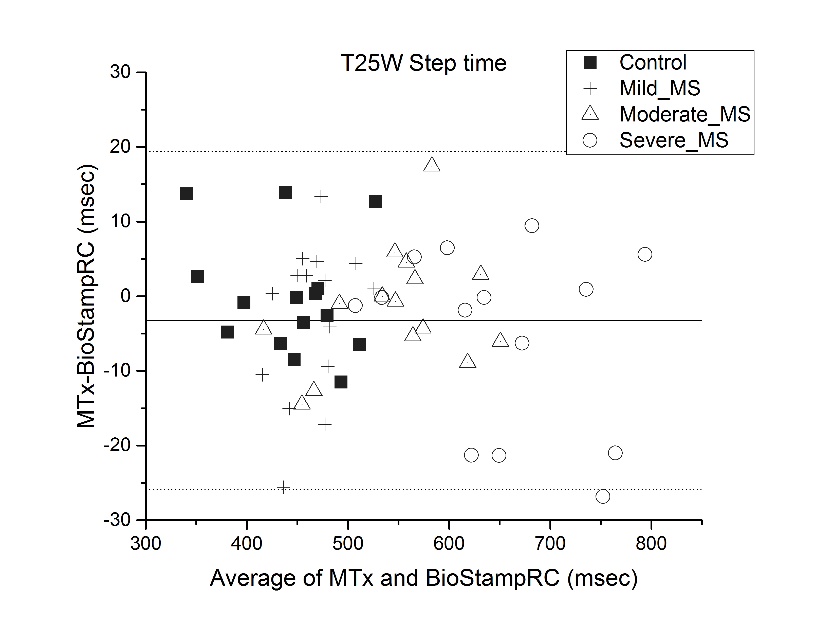


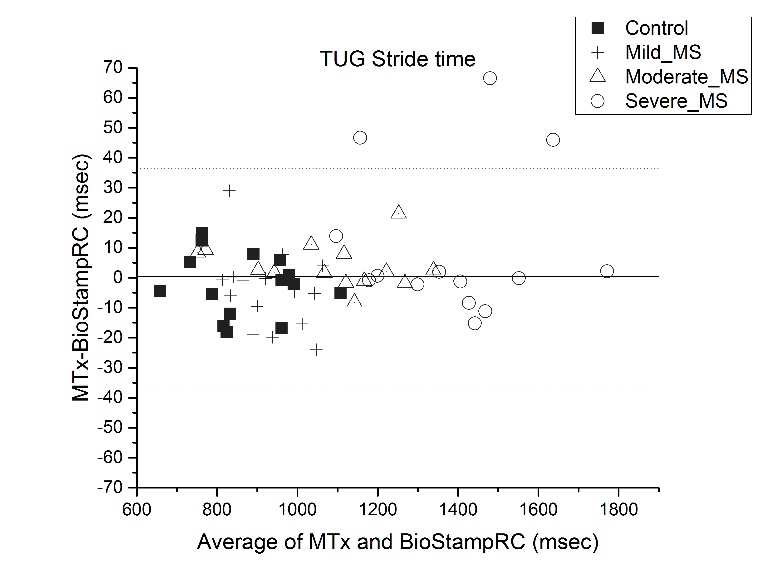


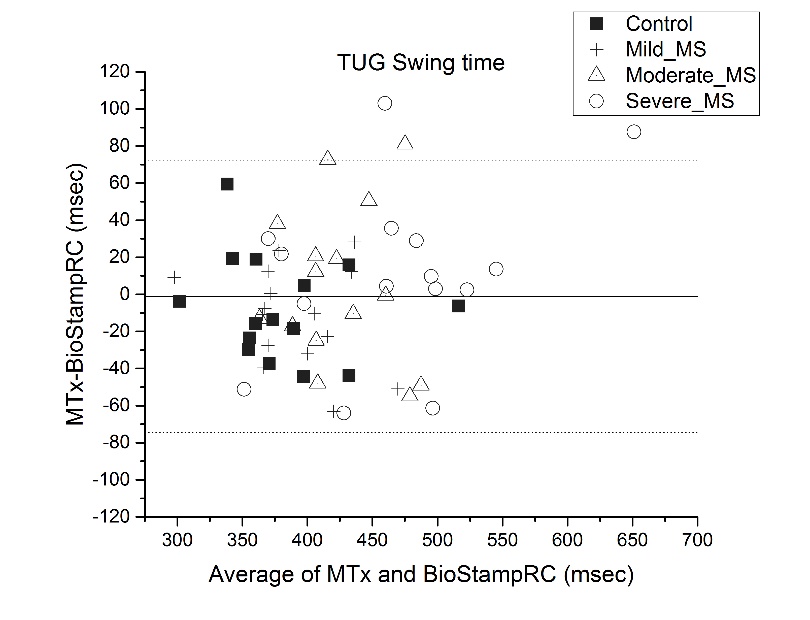


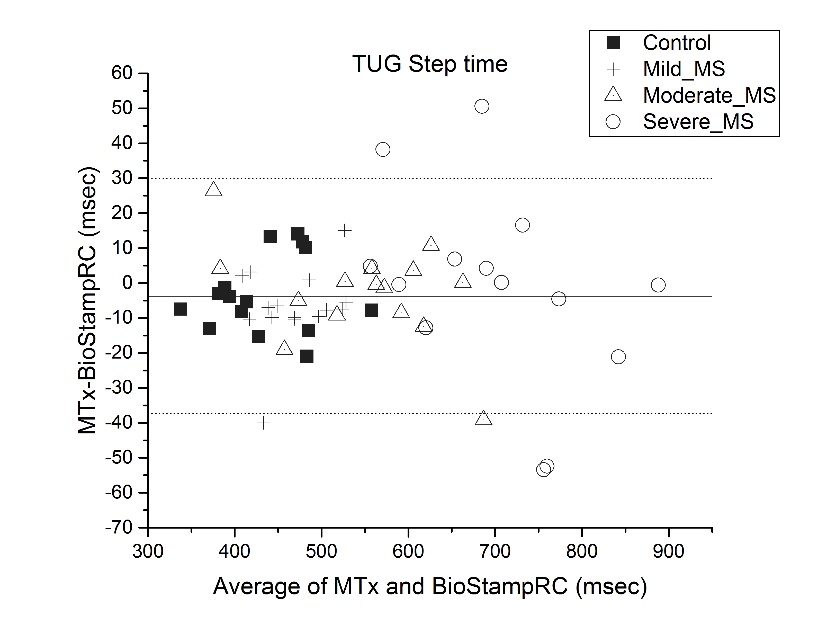


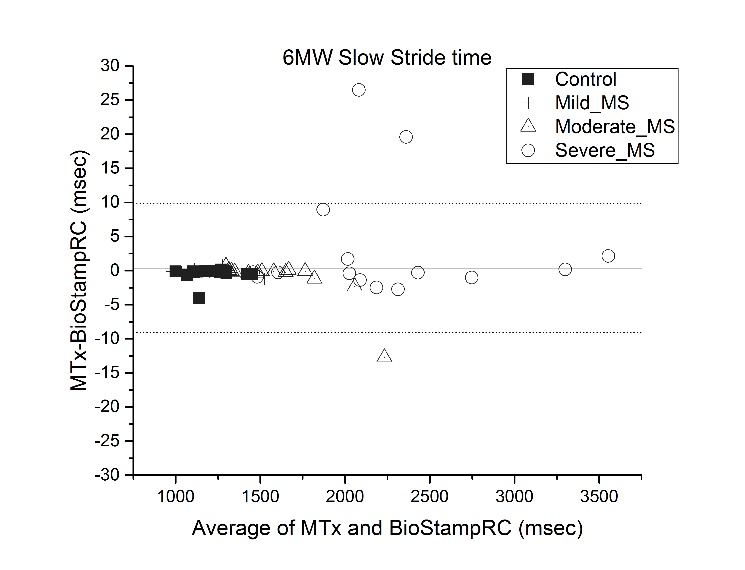


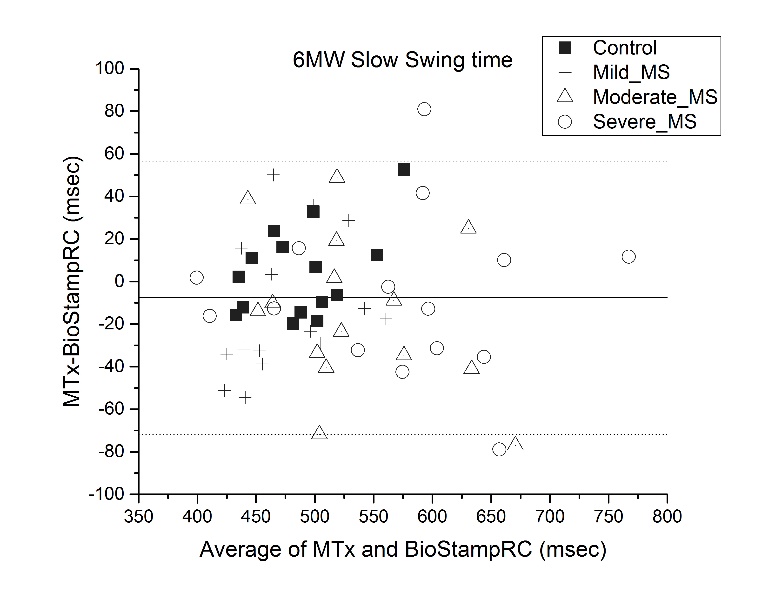


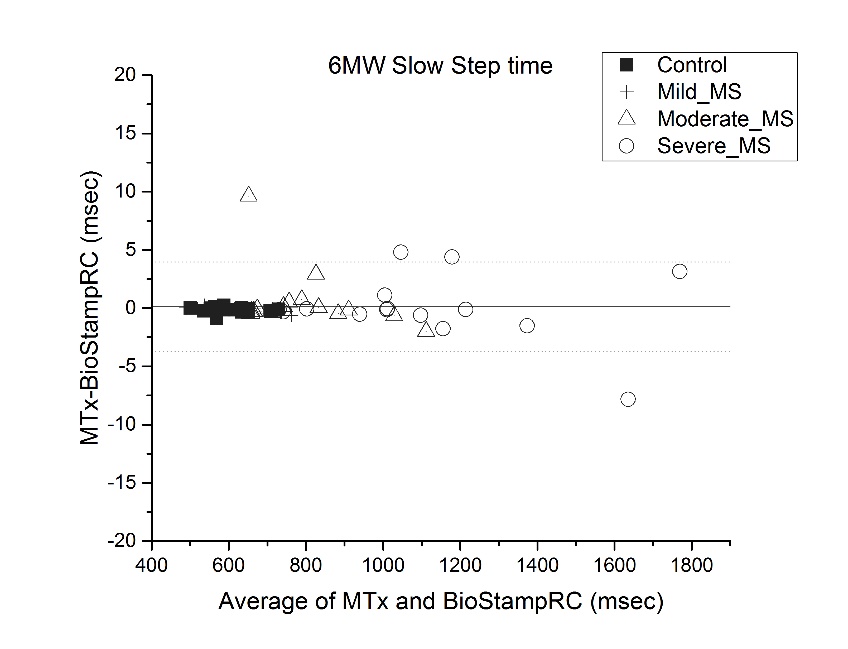


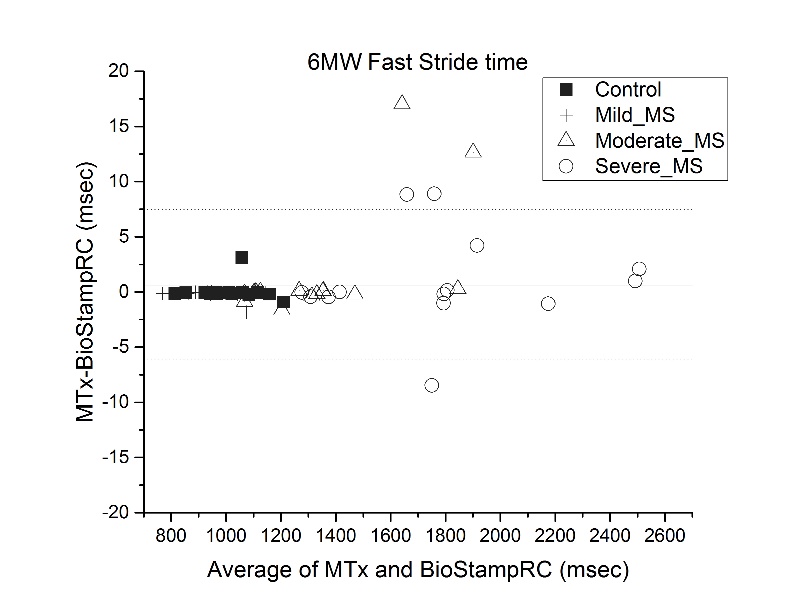


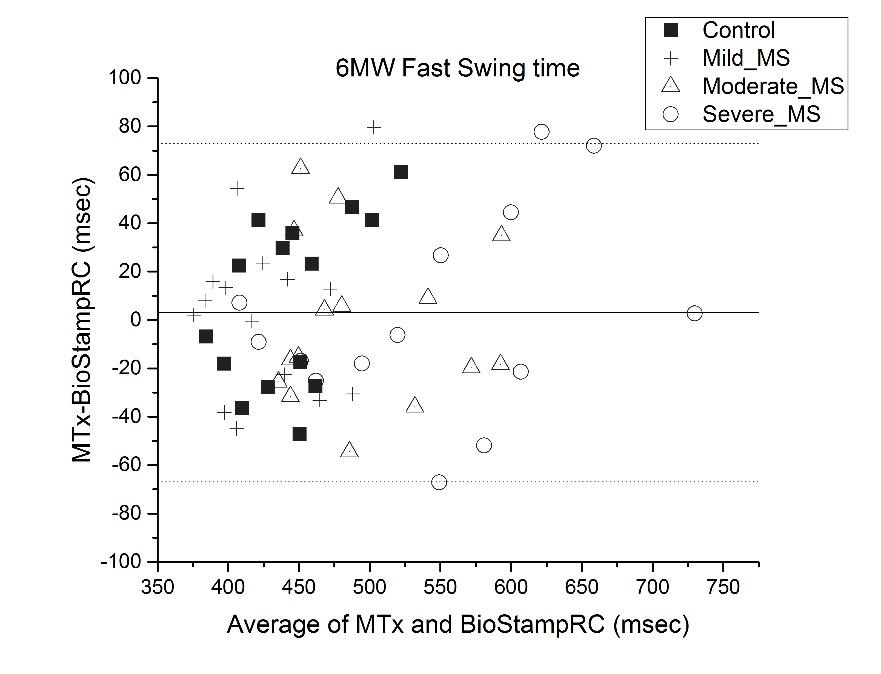


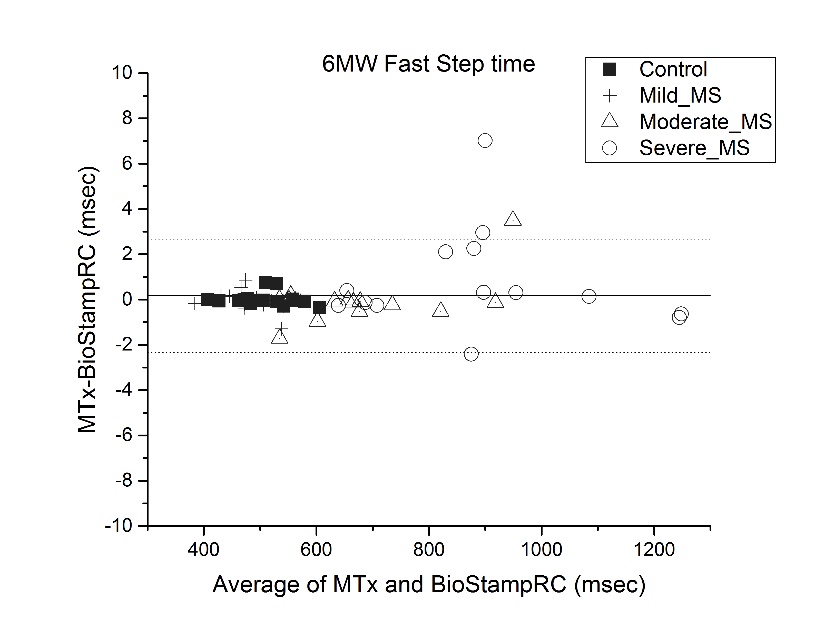

Supplement: S1 Fig — (DOCX) [file pone.0171346.s003.docx]
